# Syphilis from the Standpoints of the Syphilitic and the General Public

**Published:** 1913-04

**Authors:** Cosby Swanson

**Affiliations:** Atlanta, Ga.


### Journal-Record of Medicine

Successor to Atlanta Medical and Surgical Journal, Established 1855
and Southern Medical Record, Established 1870

**OWNED BY THE ATLANTA MEDICAL JOURNAL COMPANY**

PUBLISHED MONTHLY

*Official Organ Fulton County Medical Society, State Examining Board, Presbyterian Hospital, Atlanta, Birmingham and Atlantic Railroad Surgeons' Association, Chattahoochee Valley Medical and Surgical Association, Etc.*

EDGAR BALLENGER, M. D., Editor
BERNARD WOLFF, M. D., Supervising Editor
A. W. STIRLING, M. D., C. M., D. P. H.; J. S. HURT, B. Ph., M.D.
GEO. M. NILES, M. D., W. J. LOVE, M. D., (Ala.); Associate Editors
E. W. ALLEN, Business Manager

**COLLABORATORS**

DR. W. F. WESTMORLAND, General Surgery
F. W. McRAE, M. D., Abdominal Surgery
H. F. HARRIS, M. D., Pathology and Bacteriology
E. B. BLOCK, M. D., Diseases of the Nervous System
MICHAEL HOKE, M. D., Orthopedic Surgery
CYRUS W. STRICKLER, M. D., Legal Medicine and Medical Legislation
E. C. DAVIS, A. B., M. D., Obstetrics
E. G. JONES, A. B., M. D., Gynecology
R. T. DORSEY, Jr., B. S., M. D., Medicine
L. M. GAINES, A. B., M. D., Internal Medicine
GEO. C. MIZELL, M. D., Diseases of the Stomach and Intestines
L. B. CLARKE, M. D., Pediatrics
EDGAR PAULIN, M. D., Opsonic Medicine
THEODORE TOEPEL, M. D., Mechano Therapy
R. R. DALY, M. D., Medical Society
A. W. STIRLING, M. D., Etc., Diseases of the Eye, Ear, Nose and Throat
BERNARD WOLFF, M. D., Diseases of the Skin
E. G. BALLENGER, M. D., Diseases of the Genito-Urinary Organs

VOLUME LX | APRIL, 1913 | NO. 1

### SYPHILIS FROM THE STANDPOINTS OF THE SYPHILITIC AND THE GENERAL PUBLIC

COSBY SWANSON, M. D., ATLANTA, GA.

The advances of medical science during the past few years have been phenomenal, and especially is this true in regard to syphilis. By the persevering efforts of able scientists our knowledge of syphilis has passed through the pessimistic era, and now is on a solid foundation, one on which we have acquired a se-

W1
AT459H

cure foothold, all of which is associated with historical and technical details of no little interest and importance.

Syphilitics as a class are prone to seek information concerning their disease from "quacks," almanacs, and patent medicine advertisements, and other irresponsible sources, and it behooves us as physicians to see to it that they receive proper information.

Syphilis From the Standpoint of the Syphilitic

The syphilitic should have a general knowledge of the nature of the disease of which he is a victim. He should realize that he is suffering from a contagious, inoculable and transmissible disease, caused by a specific microorganism; chronic in course, indefinite in duration, intermittent in character, manifesting itself by a sucession of lesions which may involve any part of the body. It is no longer regarded as a mysterious disease. With the recent advances made we have fairly reliable laboratory tests to aid us in correctly diagnosing and treating the disease, making it possible to remove the unfortunate stigma.

The misapprehension that it is merely a venereal disease should be cleared up. While it is conceded that coitus is the most common mode of transmission, it is often conveyed from one person to another by direct contact of surfaces, abrasions, cracks, and non-venereal ulcerating lesions.

No disease plays such a role in transmitting itself to posterity as syphilis, and the infection may be transmitted from the father or mother, or both, but usually from the mother to the fetus through the placenta. And the frequency with which it is transmitted, the severity of its lesions, its crippling, deforming and often fatal effects, makes it an outrage for a syphilitic to marry until properly treated, and it should be made illegal; as it is impossible to conceive of any plan by which it can be otherwise prevented. At least he is due society certain precautions against infecting others. He should never kiss any one, as there is great danger of infecting the lips, tongue, and throat of innocent parties. He should avoid the use of drinking cups, knives, forks, towels, razors, brushes, etc., that others are going to use. The syphilitic woman should know that syphilis is the only known intrauterine infectious disease. Maternal transmission is more certain and more potent for harm than paternal, and the danger of its occurrence persists longer, Even though the mother is apparently free from the disease, she may be subject to repeated miscarriages or give birth to unhealthy and deformed children. This in the majority of cases is due to syphilis and should receive thorough, systematic anti-syphilitic treatment.

The necessity of early positive diagnosis is greater in syphilis than in any other disease. During its very early periods it is usually much more readily recognized by one well trained in the clinical appearance of diseases of the skin, genito-urinary organs, mouth and throat, but as soon as it enters into the chronic stages it manifests most diverse and often obscure symptoms. Here errors in the diagnosis are great and common and special skill and judgment are required.

Millions of micro-organisms multiply and spread all over the body causing the severe form that the diseas often assumes. It so lowers the resistance that complicating diseases easily occur; especially is this true when the nervous system in involved. The organs are prone to disintegrate beyond repair. Early diagnosis and treatment prevent all this; protect the community by preventing secondary lesions, mucous patches and moist condylomata, which are the most infectious manifestations of the disease, and shorten the time of treatment by many months and in some cases even years.

Later in life, should the patient exhibit any new lesions or chronic affections, his knowledge of having had syphilis is of the utmost importance in deciding as to the character of the condition and the course of treatment to be followed, and it is very important for him to be frank and open in giving his syphilic history. In no disease is it so necessary to have the co-operation of our patients as in syphilis. The patient should know that unless he carefully carries out the doctor's instructions our present methods will accomplish very little in finally curing the disease.

The treatment of syphilis is no longer the control of symptoms. The cause being known, it has at last become possible to employ a rational line of treatment, and we now have a reliable test for gauging the results of the treatment. The patient with syphilis should know that every honest scientific physician undertakes the treatment of his disease with a full appreciation of the serious nature of the infection, and carries it out as energetically as can be borne, using such remedies as possess the power of killing the causative agent.

In the treatment of syphilis it is always necessary to enforce the elementary rules of hygiene, such as regular hours, simple food, fresh air, exercise, care of the mouth and teeth, abstinence from the excessive use of tobacco, alcoholics and other stimulants, and take every possible care to keep the body in as vigorous a condition as possible.

It was, and still is believed by some that a syphilitic has an absolute immunity against syphilis that lasts from the time of the initial lesion throughout the patient's life, or until the disease is absolutely cured. However, this is not always true. It is now known that one cannot be considered absolutely immune to syphilis at any time. A super-infection may occur weeks, months or even years after the original infection. It is true that only in rare instances does the immunity resulting from a previous attack of the disease wear away, and this usually occurs only after many years.

The profession generally recognizes the prompt curative effects of salvarsan in syphilis when properly administered in sufficiently repeated doses, accomplishing in days results that it took months to produce with the old line of treatment. A false impression has been created by the extravagant claims of "quacks" and misguided enthusiasts among the profession, that two or three doses of salvarsan, given alone or in combination with a few pills, will cure the disease. In very early favorable cases it may be possible to cure the disease by this method, but it cannot be relied upon. The length of time since the introduction of its use has been too short to properly estimate the frequency of the recurrence of the disease. Nor can we depend on mercury alone to secure a final cure, no matter how long, how faithfully, or in what form we use, as is well shown by the frequent occurrence of late syphilitic conditions, such as teritary lesions, paresis, tabes, and other parasyphilitic manifestations of the disease, years after the disease has apparently been cured. In the light of our present knowledge we cannot say that syphilis is cured until the patient has been through a prolonged course of mercury with several doses of salvarsan, and repeated tests have shown that the Wassermann reaction is negative and there have been no signs or symptoms of the disease for a year after cessation of all treatment.

SYPHILIS FROM THE STANDPOINT OF THE GENERAL PUBLIC

The public demands the use of medrn scientific methods in the conservation of health, individual and national, and it is necessary for each individual to do his part in preventing the spread of this disease, which, with its complications, is one of the most common causes of death in early and middle adult life.

It is not within the scope of this contribution to discuss in detail methods of dealing with syphilis. I shall mention a few fundamental facts that should be kown by the general public, as to the mode of infection and method of prevention, etc.

If syphilis were a malady affecting the diseased individual alone it would be more permissible to allow him to live his own life as he sees fit, but as the disease is far reaching in its effects and places great burdens on society, causing the greatest economic loss to our country, by filling our poor houses and insane asylums and putting aparalytics and beggars on our streets, it is therefore one of the most important questions that we have to deal with.

It is admitted that no mtehod has as yet been devised which is in all respects unobjectionable or is capable of universal application in preventing the spread of syphilis. There is great possibility of contamination other than througgh sexual intercourse, through the use of, eating and drinking utensils pipes, musical instruments, towels, sponges, etc.; from manicurists, barbers, and chiropodists, or body contact of any kind; for example, lip and tongue chancers occur frequently as the result of kissing.

In proportion to the efficient specific measures we have at our command syphilis is one of the most poorly treated of all diseases and this is due largely to the indifference of the syphilitic.

It is a common occurrence for our periodicals and lecturers to discuss tuberculosis, cancer, typhoid fever, pellagra, and other diseases of popular interest, but the one disease, that infects a far larger percent. of the human race than any other six diseases, can only be discussed in a whisper.

It is, of course, diffcult to conceive how any syphilitic would marry, knowing of the danger of infecting his mate and offspring. However, the disease often presents latent periods, which may last for years, during which time, though, there are no outward signs of the disease, there still remain in the body infections micro-organisms which are often transmitted. The wife particularly is prone to suffer from this tate of affairs. Syphilis thus unknowinggly transmitted causes poverty, sickness and misery, by disminishing the working ability of the breadwinner, thus causing a great economic loss to family and state; prevents the primary purpose of marriage, the procreation of offspring, which is necessary for the continuation of the human species in general and the family in particular. It is evident that it lies within the interest not only of the parents, but also of the State, that the after-coming generation shall b physically, mentally and morally sound.

Physicians everywhere are required to report all other contagious diseases. New York City provides for reporting of all syphilitic patients. California requires it, and similar bills are pending in several other states.

The hygiene of syphilis is the same as the hygiene of other contagious diseases. By using our present knowledge it is possible to conduct a campaign whereby syphilis could be conquered within a comparatively short time; and to this end I would suggest the following measures:

1st. Let us cast aside our false modesty in discussing syphilis.

2nd. A rigid systematic reporting of all cases should be required.

3rd. All prostitutes should be regularly and systematically examined by competent physicians.

4th. All prostitutes should be taught practical methods of recognizing infectious syphilitic lesions.

5th. Early systematic treatment should be enforced in all syphilitics until the danger of infecting others has passed.

6th. All irresponsible persons with the disease should be isolated until properly treated.

7th. A law should be passed requiring a physician's certificate as to the health of the contracting parties before a marriage license is granted.

8th. The public should receive more general instruction as to adequate prophylactic methods.

9th. The public should know the gravity and prevalence of acquired and hereditary syphilis, and should understand that syphilis is not confined to the low and vile, but is encountered in all grades of the moral and social scale.

10th. Every married woman should be informed that, by permitting the approaches of a syphilitic husband, not only does she herself become liable to the disease, but also runs a risk of creating a being with few chances for life and fewer still for health of mind and body.

When these facts become clearly understood and generally known public health will be safeguarded, and not only will illegitimately acquired syphilis be greatly lessened, but accidental and guiltless contagion as well.

Only by general diffusion of knowledge of the disease can public opinion be prepared for the legislative measures which are so necessary for its eradication.

905-7 Empire Life Building.

### DIAGNOSTIC AIDS THE SURGERY OF THE BLADDER, KIDNEY, AND URETER

By Edgar G. Ballenger, M. D., and Omar F. Elder, M. D.,
(Atlanta, Ga.)

Few branches of surgery have made greater advances in recent years than has renal surgery, especially in regard to the accuracy in diagnosis. This accuracy has produced a corresponding reduction in the mortality. It further affords the operator, as a rule, ample assurance that the conditions are understood, the diseased part is being treated and that a kidney with good functional capacity exist on the other side. Both subjective and adjective symptoms are often misleading without the acid of the X-Ray, cystoscope and urethral catheterization. The refinements in diagnosis are of such vital importance to patients with renal and ureteral lesions that no half-way diagnosis should ever be made.

The use of the X-Ray should always be considered as a part only of the clinical picture and other supplementary facts should be supplied and a careful interpretation of all the data should be made if we are to escape the grave situations that may arise from "snap shot" diagnosis. For routine examination a twenty-four hour specimen of urine should be analyzed. The presence of blood in the catheterized specimen may be the results of traumatism caused by the passage of the catheter into the ureter. It is, therefore, evident that the control analysis should be available for comparison. Before X-ray examinations the patient should be thoroughly purged with castor oil the day before and an anema should be administered the morning of the day of the examination. The diet should be restricted to liquid diet for the preceding twenty-four hours. The patient should recline on his or her back with the thighs partly flexed upon the abdomen so as to bring the back close to the plates. Rapid plates should be used and the tube is brought as close down to the abdomen as possible. The patient sholds his breath while the exposure is being made. With first class X-ray machines the time of exposure varies from one-half to one or two seconds. The question of interpretation after the plates are developed needs more than ordinary discrimination. Cystoscopic examination should then be made and catheters introduced into the ureters. The specimens of urine collected from each side are studied separately and compared with the specimen voided before the examination was commenced. One c.c. of the phthalein solution as used by Roundtree and Geraghty should then be injected intramuscularly and the time of its first appearance in the urine noted. The promptness with which this appears in the urine and the readiness with which it is eliminated affords one of our best tests of renal functional activity. A stilet may be passed into the catheters which are then inserted to the pelvis of the kidney, or callargol may be injected for other X-Ray pictures. Care should be taken not to insert a stilet that is longer than the catheter. Overdistention with the collargol solution should be avoided. A set of stereoscopic plates aids much in locating a shadow. A phlebolith or calcareous deposit in the wall of the ureter in the wall may occupy a position so close to the ureter as to make the diagnosis uncertain. Of course all of the tests described cannot be done at one sitting nor are they necessary in every instance. The tests should be done until the clinical and other evidence indicates the character of the lesion. Phleboliths may occur singly, or in groups or rows on one or both sides and may be with difficulty differentiated from stones especially if they are shown on the side where we suspect trouble. Renal tuberculosis may show shadows simulating stone in the kidney. Braasch says that the shadows are probably caused by line-salts in caseous tubercles. The cystoscope and clinical facts in addition to the X-Ray findings usually enables one to make a differential diagnosis. Gall stones are occasionally found.

Cystoscopy.—Nitze was the first tou se cystocopy successfully. Previous to his work the efforts at looking into the bladder were crude and of little practical value. After discovery of the Edison incandescent lamp rapid development of cystocopy followed. There are two main types of cystoscopes; direct and indirect. The indirect views the object at right angle to the line vision. Many refinements in detail exist in the large number of instruments now on the market. For general use and ureteral cathterization the indirect instrument is much more satisfactory than the direct, though the latter is sometimes necessary in diagnosis of bladder lesions. Few operative procedures require more constant practice and study than cystocopy and ureteral catheterization. After once being mastered, however, there are a few things in genito-urinary surgery which afford more pleasure and satisfaction than does the use of the cystoscope. The accuracy it affords and additional information gained by its use make this branch of medicine and surgery second to none in scientific exactness. Persistent bladder and renal affections which suggest stone, tumor sacculations, kinks in the ureters, beginning or well established hydronephrosis or pyonephrosis require cystoscopic examination, ureteral catherization, pyelography, X-Ray examinations, etc.

Technic.—Unless one is familiar with the use of the cystoscope he should study well the mechanics of his insturment as well as its practical use on phantom bladders. Squier suggests the use of a hollow rubber ball a little larger than a tennis ball, this is attached to a block of wood; openings are made to represent the urethra and ends of the ureters. By the study of such a phantom many points in the technic may be worked out.

Sterilization of the Instruments.—The cystoscopes, of which the Brown-Buerger is the best, should be thoroughly washed with soap and water and immersed for one hour in lysol 1-30, or carbolic 1-30. They then should be transferred to sterile water before using. The patient is placed in a semisitting posture with the hips near the end of the table and the thighs fixed on the trunk and held in "rests" attached to the table. The trousers and drawers of male patients should be removed; the penis, pubic region and scrotum should be well scrubbed, then surrounded with sterile sheets and towels. The urethra should be filled with a cocainized tragacanth lubricant which should be pressed into the deep urethra. Care is taken to try again the light to see if it is working properly. The cystoscope is then introduced just as a sound would be. The obturator is removed and the bladder is irrigated with warm sterile boric acid solution until it returns clear. A specimen of the urine voided before the operation is started should always be preserved for comparison with specimens that may be secured with blood, which of course may be traumatic. The desired telescope with or without the ureteral catheters is then inserted and 180 to 240 c.c. (6 to 8 ounces) of boric acid solution is injected through the irrigating cock. With the light turned on we now begin the examination. Naturally one must be familiar with the appearance of the normal bladder before pathologic conditions can be recognized. It should be remembered that with the indirect cystoscope the picture as seen is inverted, unless a correcting lens is provided. One should try as early as possible to get his bearings by finding some point as the vesical sphincter, ureteral opening, or the bubble of air in the dome of the bladder. As the instrument is withdrawn a dark red area is seen; this is the neck of the bladder. By advancing, rotating, elevating and depressing the cystoscope the entire bladder wall may be reviewed. The ureteral openings appear as two small slits on each side and are seen when the cystoscope is rotated about 30 degrees. It is in the trigone that we usually find the most important things, such as stones, ulcers and tumors. The character of the mucous membrane around the ureteral openings often give a clue as to the probable lesion. Tuberculosis of the bladder or the reddened ureteral orifice, with tubercles or ulcerations, presents a striking and diagnostic picture. Trabeculations of the bladder wall may be recognized, as also saculations. Tumors of the bladder may be readily seen. Blood clots and mucus may be mistaken for such tumors as pappillomata. Foreign bodies will occasionally be seen.

Roundtree and Geraghtys Phenolsulphonephthalein Test of the Functional Capacity of the Kidneys.—This is the best of the tests to determine the functionating power of the kidneys. Taken in connection with the tests for albumin, casts, chromocystoscopy with indigocarmine, specific gravity and the rapidity with which the quality of urine increases when an additional amount of water is consumed, we are enabled to ascertain with more than ordinary accuracy the condition of the kidneys. We utilize this phthalein test in all instances where we wish to know the condition of the kidneys to anticipate and thus minimize the danger of the post-operative anuria by the application of appropriate measures before the operation. If the condition is non-operative we think this test affords a valuable guide in making the prognosis in Bright's disease. Where we expect to operate upon the kidney we catheterize the ureters and apply this test to each kidney separately and thus far it has not mislead us to thinking a kidney had good functional capacity when it did not. The test deserves a much more general adoption on account of both its value and its simplicity. Geraghty who was one of the originators of the test, describes it as follows and gives his results after an extensive application in many renal affections:

"The technic of estimating total function without the use of urethral catheters is as follows: One c.c. of phthalein solution (6 mg) is injected deep into the muscles, preferably the lumbar muscles, and the patient instructed to void at the end of one hour and ten minutes, and again at the end of two hours and ten minutes from the time of the injection, each specimen being kept separate. The drug should appear normally in about ten minutes, and this much time is consequently allowed for its appearance. In this method no account is taken of the time of appearance, the main reliance being placed on the quantity excreted. This method is extremely simple.

"In cases with urinary obstruction a catheter must be employed. The time of appearance is estimated in these cases, the collection being made for two separate hours from the time of appearance of the drug in the urine. When the phthalein excretion is high for the first hour it is unnecessary to continue the test longer. The estimation of the amount of drug excreted is made by means of a modified Hellige colorimeter. The estimations can be made quite accurately for practical purposes by means of two graduated test-tubes. When the test is used in conjunction with urethral catheterization the injection is made intravenously, and the urine collected for two separate fifteen-minutes intervals, the time of collection beginning with the appearance of the drug on the first side. When the kidneys are functioning smoothly, and the function is being measured simply to determine the efficiency of the remaining kidney, should nephrectomy be performed, a collection for fifteen minutes is usually all that is necessary. If the second kidney is normal, a high excretion of phthalein will occur, sufficient to indicate its efficiency. When however, both kidneys are diseased or when the functional estimations are being made for purposes of diagnosis, comparison of the two kidneys being desired, periods longer than fifteen minutes must be employed. For short periods the kidneys normally vary much in the relative amount of work which each one performs, but if the time of collection is one hour the variation will be slight. When very accurate relative estimations are desired intramuscular injections and collection for one hour should be made. The total function without the use of the ureteral catheters is always determined in these cases for purposes of comparison with the separate function and to check up the amount of catheter inhibition, should this occur.

"The test has been employed now in over 20 cases of nephritis of varying types, approximately 350 cases of urinary obstruction, mostly prostatic cases, in 150 cases of unilateral or bilateral disease in conjunction with a thousand other cases as part of a routine examination. Our added experience confirms entirely our early conclusions regarding the reliability and accuracy of the test. In the milder form of so-called glomercular or parenchymatous nephritis the definite clinical evidence of renal disease seemed at first irreconcilable with the relatively high phthalein output. Phthalein is eliminated almost if not entirely by the tubules, so the presence of glomerular disease would not necessarily cause decreased phthalein elimination. The glomeruli, however, are rarely severely diseased without considerable resultant damage to the tubules, and the greater the tubular injury the more marked the phthalein decrease. In these cases with disease of long standing, the elimination is markedly decreased. In the chronic interstitial type of nephritis individuals with low phthalein excretion are occasionally seen in whom no evidence of uremia is present, and there is somewhat of a tendency for this reason to doubt the accuracy of the test. There have been some such cases in our series, and in these death resulted some months later from uremia. Autopsy in each case proved the presence of a very advanced chronic nephritis. In the routine examination of patients, one was found whose excretion of phthalein was 7 per cent. for two hours, although no suspicion of any renal disease was entertained in this case. The most careful examination failed to give any evidence of the existence of a nephritis. The patient was kept under observation, and a few months later suddenly developed uremia which ended in death. Autopsy showed an extreme grade of chronic interstitial nephritis.

"In thirty-five cases the opportunity has presented itself of verifying the accuracy of the test at autopsy. The lesions as revealed correspond closely with the efficiency, as shown by the test. In one case with a practically normal output, a moderate grade of double polycystic kidney was found. The urine itself showed no abnormalities, and the condition was unsuspected. In thirty cases a nephrectomy has been performed and an opportunity afforded of comparing the pathologic picture with the function, as shown before operation. The phthalein excretion and the amount of kidney destruction seemed to correspond in a striking manner. It would be well to remind those using phthalein test that it is only a test of the excreting power of the kidney, and not a test of the normal or abnormal function of other organs. It furnishes, when taken in conjunction with the ordinary clinical examination, accurate information which can be obtained at the present time in no other way. With added experience we find no reason to change our previous views as regards the accuracy of the test in measuring the excretion power of the kidneys."

Technic of Making X-Ray Picture of the Kidney Region.—A good plate depends upon many factors. Derr describes as follows his technic:

"A good machine, a tube of the proper vacuum, a skillful technic and the proper development and interpretation of the plate after it is obtained. The preparation of the patient may also be an important factor in the result, as masses of feces and bubbles of gas in the intestines destroy the clearness of the X-ray negative, and the former may even simulate a stone. The intestinal tract should be thoroughly cleared with castor oil and food should be sparingly taken of before the examination. This is made with the patient in the dorsal position and the lumbar curve brought down to the table as closely as possible, the plate being placed beneath, and extending from the 10th rib downward to the iliac crest. On very stout subjects the abdominal binder will be found useful to reduce the thickness of the abdomen. In all cases the compression diaphragm of Albers-Schonberg with hemispherical aluminum window is of great advantage to push the intestines out of the path of the rays. The cone should be tilted at such an angle as to throw the line of focus upward beneath the costal border, if possible; the degree of the angle to be suggested by the anatomy of the patient. By this procedure the kidney is penetrated diagonally and its shadow is brought out with the greatest possible distinctness, the distortion produced being very trifling. Unless the patient is small, I find it best to make a separate focus and exposure for each kidney.

"For examining the ureter for stone, the focus of the tube should be directed downward at a considerable angle in order to project the shadow through the pelvic canal and on the plate. My apparatus is a 4 k. w. Wate & Bartlett Transformer, running at 110 volts, direct current. The character of Roentgen tube should be such that 40 to 50 milliamperes of current can be passed, using the full power. The softer the tube the better, provided it has the necessary penetration. This tends to bring out the greatest amount of detail in soft structures, such as the psoas muscle, liver and spleen. Too hard a tube too long an exposure may penetrate the calculus.

"The time of exposure varies according to the thickness of the subject from two to five seconds by time switch. Such a technique renders it possible to make as many exposures as may be desired, without the slightest danger of injury to the patient. In the early days of the X-ray, it was not uncommon to expose for 30 to 40 minutes in efforts to penetrate the abdomen, and a case is on record of a woman who, after three such exposures developed a sloughing wound from which she died. The dread of the X-ray burn still persists in the minds of many who are not familiar with the advances made in modern technic. The exposure is made while the patient holds his breath, either in expiration or inspiration; both have their advantages in particular cases, though as a rule, the former is the best, as the kidneys move downward with the diaphragm and the costal margins are more elevated."

THE CATHETER STILET.—Kolischer and Schmidt first suggested the introduction of a ureteral catheter containing a wire stilet, hoping thus to identify doubtful ureteral and kidney shadows. While this method is of value it is not infallible even when the radiograph is taken at different angles. Looking at the plates through a stereoscope so as to apparently add depth aids in the localization of shadows. Instead of the stilet the catheter may be filled with a saturated solution of colloidal silver and then introduced. This is even better than the stilet as a kink in the ureter is less likely to be obliterated. If the catheter meets an obstruction in the ureter and the urine flows freely after it passes, the probability is that the suspected shadow is a stone. To obtain the best results in the diagnosis there should be close co-operation and team-work between the surgeon, the cystoscopist and the radiologist.

CHROMO-URETEROSCOPY.—Voeleker and Joseph devised this test using indigo-carmen. We think it comes second to the phthalin test though each has certain advantages the other does not possess. Both should be used when needed. There are times when it is inadvisable to catheterize the ureters to determine the functional capacity of the kidneys; in such instances the indigocarmin test is of value. It has a further advantage of not producing a mechanical action as sometimes does the ureteral catheter in temporarily causing a stimulation or suppression of the renal function. The urethral catheter may not catch all of the urine and the part escaping into the bladder may cause an erroneous deduction unless a control test is made of the fluid in the bladder. In this respect the indigocarmin test has an advantage. Chromo-ureteroscopy is simpler because the ureters do not have to be catheterizzed in making a unilateral determination.

THE TECHNIC OF THE INDIGOCARMIN TEST.—The patient is prepared as usual for cystoscopy. Four c.c. of 4% of indigocarmine is injected intravenously or into the gluteal muscles, the exact time being carefully noted. The openings of the ureters are then watched until blue urine is seen to flow from them. Thomas found that indogocarmin is eliminated from functionally sufficient kidneys as a dark blue in from three to twenty minutes, and as a light blue not later than fifteen minutes. In 90 per cent. of kidneys it appeared either as a dark or light blue in fifteen minutes; in .61 per cent. it was observed in ten minutes. He also observed that morphine and ether delayed the appearance of the dye. The intensity as well as the time of the appearance should both be noted and recorded. A delay of more than 20 minutes means renal insufficiency. Regarding this Thomas says: "although the exact time limit for functionally sufficient kidneys has probably not yet been definitely determined, my experience has indicated that, in the event of the excretion of the dye as a dark blue, it should be placed, for the present at least, at twenty minutes; while if the elimination occurs as a light blue, fifteen minutes should be the time limit. This applies strictly to prognosis in view of nephrotomy, the figures will vary and no time limit can at present be stated. In nephrectomy likewise surgical technic and skill, proper selection and administration of anesthetics, postoperative treatment and renal reflexes play a most important role with respect to recovery. These are variables which no functional kidney test will ever gauge. The onset of the excretion of the dye as a light blue after fifteen minutes and continuing as such undoubtedly indicates functional degeneration of the respective kidney. Precisely at what minute the ultimate time limit for function sufficiency of such a kidney expires, is at present undetermined." Each urethral orifice should be observed alternately to see the differences in the intensity of the color.

Pyelograpy.—Voelker, in 1906 demonstrated that a solution of collargol injected into the ureter and pelvis of kidney would produce a shadow on the X-ray plate. Three years later Braasch applied this method at St. Mary's Hospital, and has now used it in more than 600 cases. Many others have confirmed its value. The method is not entirely devoid of danger if care is not exercised in the selection of the patients or if too great pressure be used in injecting the solution. Collargol 15% or argyrol 25% will either produce a shadow though the former is better. In this manner we may outline the lumen and the cavities of the ureters and kidney, and thus show their normal or abnormal characteristics. The ureters are first catheterized and then the solution is injected carefully so as not to overdistend the pelvis. The X-ray picture is immediately taken—previous purgation and restriction of the diet having been carried out. Its chief value lies in its showing us the extent and character of dilation of the ureter or renal pelvis; deformity accompanying renal tremors; and congenital anomalies. Braasch says that the majority of instances of stone in the ureter there will be more or less urethral diltation above it, provided the injected medium can pass the obstruction. He thinks that when the injected ureter shows no dilation above a doubtful shadow we can as a rule, infer that the shadow is not due to a stone. Braasch says further that it has been their experience at the Mayo clinic that a distinctly abnormal pelvic outline or other evidences of tumor involvement can be demonstrated in the pyelograph in the majority of renal tremors. The pelvic changes must be well marked in order to be recognizable. "They will consist of —(1) Retraction of individual calices, presenting stricking bizarre outlines; (2) irregular distention of the entire pelvis; and (3) obliteration of the pelvic lumen partial or complete." Retraction of the upper ureter by surrounding renal neoplasm may be confirmatory evidence. The reiation of the injected pelvis to an unidentified tumor may also be of differential value. In our own experience with pyelography we had no ill effects following its use and have obtained much valuable information from its application in those instances where additional facts were necessary to complete the diagnosis."

